# Favorable effects of low-fat and low-carbohydrate dietary patterns on serum leptin, but not adiponectin, among overweight and obese premenopausal women: a randomized trial

**DOI:** 10.1186/2193-1801-3-175

**Published:** 2014-04-04

**Authors:** Adana AM Llanos, Jessica L Krok, Juan Peng, Michael L Pennell, Susan Olivo-Marston, Mara Z Vitolins, Cecilia R DeGraffinreid, Electra D Paskett

**Affiliations:** Division of Population Sciences, The Ohio State University Comprehensive Cancer Center, Columbus, OH USA; Department of Epidemiology, RBHS-School of Public Health, Rutgers University and the Rutgers Cancer Institute of New Jersey, New Brunswick, NJ USA; Division of Biostatistics, College of Public Health, The Ohio State University, Columbus, OH USA; Division of Epidemiology, College of Public Health, The Ohio State University, Columbus, OH USA; Division of Cancer Prevention and Control, College of Medicine, The Ohio State University, Columbus, OH USA; Department of Public Health Sciences, Wake Forest School of Medicine, Winston-Salem, NC USA; The Ohio State University Comprehensive Cancer Center, 1590 N. High St., Suite 525, Columbus, OH 43210 USA

**Keywords:** Breast cancer prevention, Dietary intervention trial, Low-fat diet, Low-carbohydrate diet, Premenopausal women, Adipokines, Adiponectin, Leptin

## Abstract

**Purpose:**

The most effective dietary pattern for breast cancer prevention has been greatly debated in recent years. Studies have examined hypocaloric diets, with particular emphasis on macronutrient composition, yielding inconclusive data. The objective of this study was to examine the effects of calorie-restricted low-fat and low-carbohydrate diets (LFD and LCD, respectively) on circulating adipokines among overweight and obese premenopausal women.

**Methods:**

Seventy-nine overweight and obese premenopausal women were randomized to either LFD or LCD, with increased physical activity, for 52 weeks. Serum adiponectin, leptin and the adiponectin-to-leptin ratio (A/L) were measured at baseline, and at weeks 34 and 52 to assess intervention effects.

**Results:**

While there were no significant changes in serum adiponectin concentrations following the LCD and LFD interventions, leptin concentrations significantly decreased by week 34 of the intervention period (LCD: 35.3%, *P* = 0.004; LFD: 30.0%, *P* = 0.01), with no difference by intervention arm. At week 52, these reductions were statistically non-significant, indicating a return to baseline levels by the end of the intervention. While there were non-significant increases in the A/L ratio following the LCD and LFD intervention arms, the overall trend, across groups, was marginally significant (*P = 0.05*) with increases of 16.2% and 35.1% at weeks 34 and 52, respectively.

**Conclusions:**

These findings suggest that caloric-restricted LCD and LFD dietary patterns favorably modify leptin and possibly the A/L ratio, and lend support to the hypothesis that these interventions may be effective for obesity-related breast cancer prevention through their effects on biomarkers involved in metabolic pathways.

**Trial registration:**

Clinical Trial Registration Number: NCT01559194.

## Introduction

Epidemiologic studies suggest that the modern lifestyle of developed countries, characterized by high levels of physical inactivity and high caloric intake, leads to greater adiposity and potentially increases the risk of breast cancer (Calle et al. 2003; Byers et al. 2002). Proposed explanations for this association include involvement of the obesity-related adipokines, adiponectin and leptin (Siiteri 1987), although, among premenopausal women, specifically, the data supporting a breast cancer association for adiponectin (inverse) (Minatoya et al. 2013; Liu et al. 2013; Ye et al. 2014; Tian et al. 2007; Tworoger et al. 2007; Mantzoros et al. 2004; Miyoshi et al. 2003) and leptin (positive) (Wu et al. 2009; Liu et al. 2007; Mantzoros et al. 1999; Tessitore et al. 2000; Woo et al. 2006; Harris et al. 2011; Petridou et al. 2000; Falk et al. 2006) are inconsistent.

While among postmenopausal women there is strong evidence for a positive association between obesity and breast cancer risk (Carmichael and Bates 2004), evidence to support this association among premenopausal women is not currently available. Nonetheless, breast cancer outcomes are substantially worsened as a result of obesity irrespective of menopausal status (Carmichael and Bates 2004). Thus, while data suggest reduced breast cancer risk among obese premenopausal women, excess weight and weight gain during adulthood, particularly after the onset of menopause, ultimately increases their risk. Several modifiable behaviors (*e.g.*, diet and physical activity [PA]), which could effectively prevent obesity and favorably modify circulating adipokine concentrations, may be paramount to both cancer prevention and improvement of outcomes following a breast cancer diagnosis.

The most effective dietary pattern for breast cancer prevention is a controversial topic and greatly debated among various disciplines in the scientific community. Although most studies have focused on low-fat diets (LFDs), few have investigated the optimal protein and carbohydrate intake for breast cancer prevention (Sieri et al. 2002; Toniolo et al. 1989; Martin-Moreno et al. 1994). Proponents of low carbohydrate diets (LCDs) report that high carbohydrate intake results in higher plasma insulin levels and promotes lipogenesis (Bilsborough and Crowe 2003); hence the popularity of low-carbohydrate diets (*e.g.*, the Atkins diet) among the general population. Some short-term studies (*e.g.*, ≤6 months) of LCDs have reported greater loss of body fat and greater maintenance of lean body mass when compared with diets high in carbohydrates (Farnsworth et al. 2003; Foster et al. 2003). These studies have been relatively short-term, and, therefore, the effect on long-term weight maintenance is unknown.

In this study, we examined the effects of two calorie-restricted diets (LFD and LCD) plus PA on serum concentrations of adiponectin, leptin and the adiponectin-to-leptin (A/L) ratio in overweight and obese premenopausal women. We hypothesized that diets low in fat and carbohydrates would favorably improve serum adipokine profiles, specifically by increasing adiponectin and decreasing leptin concentrations, as a result of diet-induced reductions in anthropometric measures (*e.g.*, weight and body mass index [BMI]).

## Methods

Premenopausal women were recruited for a 52-week randomized intervention trial of two calorie-restricted dietary patterns plus PA. The diets were: 1) LFD: 20% of total calories from fat, 20% from protein and 60% from carbohydrates); and 2) LCD: 40% of total calories from carbohydrates, 30% from protein and 30% from fat). All study participants were educated on caloric-restriction and given a personalized PA prescription. Women were randomized using stratified randomization, based on BMI (<30 kg/m^2^ vs. ≥30 kg/m^2^) to ensure equal distribution of overweight and obese participants into each intervention group. This study received ethical approval from the Cancer Institutional Review Board at the Ohio State University.

Screening and recruitment occurred between May 2005 and August 2006, at primary care physician’s offices and through media advertisements. Potential participants called a designated telephone number and a staff member from the Comprehensive Cancer Center provided information about the study and conducted eligibility screening. Women who: were ≥30 years and premenopausal (confirmed by follicle stimulating hormone concentrations); had no prior cancer diagnosis; BMI 25–34 kg/m^2^; were residents of the Columbus, OH area during the 18 month follow-up period; and obtained medical clearance for participation in PA (from their primary care physician), were eligible to participate. Women who: were pregnant or planning to become pregnant; were enrolled in a structured weight loss program; had medical condition(s) precluding adherence to the dietary interventions; and/or had uncontrolled existing medical conditions, were ineligible from study participation. All participants provided written informed consent prior to study enrollment.

### Intervention

The dietary education provided to study participants was based on the Exchange System for weight management (Franz et al. 1987). A registered dietician (RD) assessed the level of caloric-restriction that would be required based upon a participant’s resting metabolic rate, which was determined from indirect calorimetry at baseline. Once the required caloric-restriction was determined, the RD developed a plan of servings (“exchanges”) from each food group that would fulfill the macronutrient distribution required for the diet assigned at randomization. After baseline measures were assessed, each participant met with an RD at the university’s Clinic Research Center (CRC) once per week for the first month of the study period, every three weeks during the second, third and fourth months of the study, and every 6 weeks for the remainder of the study, except weeks 34 and 52, which were clinic visits. If a participant was unable to attend a meeting with the RD, sessions were conducted by telephone.

Adherence to the dietary interventions was assessed through 7-day dietary recalls. Participants documented their dietary consumption for the first 7 days of each month during the study and submitted dietary recalls for analysis during their next visit. Dietary recalls were collected 12 times during the intervention period. The Food Processor software (ESHA Research, Salem, OR) was used to analyze dietary recalls and to provide personalized feedback.

Participants documented their PA (total steps per day) by wearing a pedometer (Digiwalker^TM^, Yamax Health & Sports, Inc., San Antonio, TX). Each woman was given personalized PA counseling, with the ultimate goal of 10,000 steps per day. Women were also encouraged to modify their lifestyle (*e.g.*, use stairs more frequently, park further away from destination) to facilitate reaching daily step goals. Additionally, women documented their participation in other activities in PA logs.

### Assessments

Following the initial screening, clinic visits (baseline, week 34, and week 52 [end of the study period]) to the CRC were required for anthropometric measurements and fasting blood draws. At baseline, self-reported characteristics (*e.g.*, demographic, tobacco and alcohol use, PA, quality of life data) were collected; and height, weight, and waist and hip circumference were measured by CRC staff.

### Adipokine analyses

Serum specimens, collected at baseline and at weeks 34 and 52, were used to measure changes in biochemical endpoints including adiponectin and leptin concentrations. Serum adiponectin and leptin were determined using the Human Leptin Quantikine and Human Adiponectin/Acrp30 Quantikine ELISA kits (R&D Systems, Minneapolis, MN) according to manufacturer’s instructions. Samples were assayed blindly, in duplicate, random order. Each batch included replicates, commercial controls, and blinded serum controls to assess laboratory variation. The coefficients of variation (CVs) for the serum assays were 9.18% and 6.31% for leptin and adiponectin, respectively. Assay sensitivity was <7.8 pg/mL for leptin and 0.08 ng/mL for adiponectin. No samples were below the limits of detection.

### Statistical analyses

Change in serum adiponectin, leptin, and the A/L ratio over weeks 1–52 was examined for each intervention arm, as well as the change averaged across the two diets. Linear mixed models were used and included fixed effects of treatment arm, time, and a treatment-by-time interaction. The mixed models assumed an autoregressive correlation structure for residual errors, which was implemented using the REPEATED statement in SAS PROC MIXED with covariance type = SP (POW) (SAS Institute v. 9.2, Cary, NC). The smallest Bayesian Information Criterion was used to choose between a linear, quadratic, or cubic trend in time. Measurements collected after week 34 but before week 56 were included in the analyses to allow flexibility in timing of the final follow-up. In secondary analyses, change in leptin, adiponectin, and the A/L ratio was examined based on participants’ adherence to diet or PA. Women were considered diet-adherent if their fat or carbohydrate intake was within 80% of the intervention goal (LFD: 20% of total calories from fat; and LCD: 40% of total calories from carbohydrates) according to their final diet record. Women were considered PA-adherent if their average daily steps were within 80% of the PA goal. Adiponectin, leptin, and the A/L ratio were natural log-transformed to produce residuals that were approximately normally distributed. The Kenward-Roger method for computing degrees of freedom (Kenward and Roger 1997) was used for all hypothesis tests. A two-sided significance level of α = 0.05 was used for all tests.

## Results

### Participants

**Figure 1:**
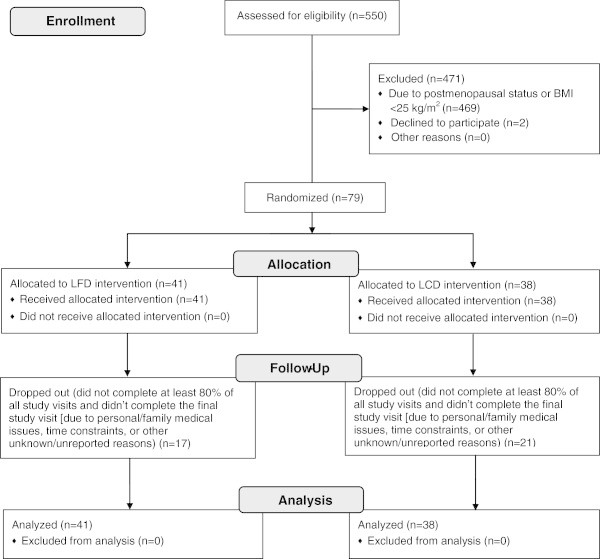
**Consort 2010 flow diagram for the randomized dietary intervention trial of low-fat and low-carbohydrate diets.**

Among the 550 women who were screened, 81 were deemed eligible for study participation. Of those, 79 women agreed to participate; 41 were randomized to the LFD arm and 38 were randomized to the LCD arm (Figure 1). Reasons for ineligibility included having a postmenopausal status or a BMI <25 kg/m^2^. Baseline characteristics of the participants included in the present analyses are shown in Table 1. Adherence to the dietary interventions was low for both arms (22% and 29% for LFD and LCD, respectively [data not shown]). Overall, participants were more compliant with the PA component of the intervention (66% and 61% among those randomized to the LFD and LCD arms, respectively [data not shown]). Additionally, a large proportion of participants dropped out (did not complete at least 80% of all study visits and didn’t complete the final study visit) with no significant difference by intervention arm (41% [n = 17] and 55% [n = 21] for LFD and LCD, respectively).

**Table 1:** **Baseline characteristics of study participants, N = 79**

	Total, N = 79 n (%)	Dietary intervention arm	
		Low-fat, n = 41 n (%)	Low-carbohydrate, n = 38 n (%)
Age (years)			
30–34	12 (15)	7 (17)	5 (13)
35–39	18 (23)	10 (24)	8 (21)
40–44	25 (32)	14 (34)	11 (29)
45–49	19 (24)	9 (22)	10 (26)
50–54	5 (6)	1 (2)	4 (11)
Race			
White	54 (68)	29 (69)	25 (68)
Black	21 (27)	11 (26)	10 (27)
Asian	1 (1)	1 (2)	0 (0)
American Indian	1 (1)	0 (0)	1 (3)
Other	2 (3)	1 (2)	1 (3)
Marital status			
Married	65 (82)	35 (83)	30 (81)
Divorced/separated	4 (5)	3 (7)	1 (3)
Single, never married	7 (9)	2 (5)	5 (14)
Single, living as married	0 (0)	0 (0)	0 (0)
Widowed	2 (3)	2 (5)	0 (0)
Unknown	1 (1)	0 (0)	1 (3)
Education			
High school diploma/GED	3 (4)	1 (2)	2 (5)
Vocational/training school	2 (3)	2 (5)	0 (0)
Some college	14 (18)	11 (26)	3 (8)
Associate’s degree	7 (9)	3 (7)	4 (11)
Bachelor’s degree	20 (25)	9 (21)	11 (30)
Master’s degree	9 (11)	4 (10)	5 (14)
Doctorate degree	4 (5)	2 (5)	2 (5)
Unknown	20 (25)	10 (24)	10 (27)
Employment status			
Full-time	38 (48)	24 (57)	14 (38)
Part-time	9 (11)	3 (7)	6 (16)
On medical leave (employed)	1 (1)	1 (2)	0 (0)
Self-employed	6 (8)	4 (10)	2 (5)
Homemaker	5 (6)	0 (0)	5 (14)
Unknown	20 (25)	10 (24)	10 (27)
Occupation			
Professional/technical-managerial/administrative	43 (54)	20 (48)	23 (62)
Sales/clerical service	7 (9)	5 (12)	2 (5)
Craftsman/machine operator/laborer	1 (1)	1 (2)	0 (0)
Other	8 (10)	6 (14)	2 (5)
Unknown	20 (25)	10 (24)	10 (27)
Annual household income			
<$10,000	1 (1)	1 (2)	0 (0)
$10,000-$19,999	2 (3)	0 (0)	2 (5)
$20,000-$34,999	4 (5)	3 (7)	1 (3)
$35,000-$49,999	7 (9)	3 (7)	4 (11)
$50,000-$74,999	12 (15)	6 (14)	6 (16)
$75,000-$99,999	15 (19)	5 (12)	10 (27)
$100,000-$149,999	10 (13)	6 (14)	4 (11)
>$150,000	5 (6)	3 (7)	2 (5)
Don’t know	1 (1)	1 (2)	0 (0)
Refused	3 (4)	2 (5)	1 (3)
Unknown	22 (28)	12 (29)	10 (27)
Health insurance status			
Private insurance	54 (68)	28 (67)	26 (70)
Military of VA insurance	1(1)	1 (2)	0 (0)
Medicare	1 (1)	1 (2)	0 (0)
Uninsured, self-pay	3 (4)	1 (2)	2 (5)
Uninsured, no means to pay	1 (1)	1 (2)	0 (0)
Unknown	19 (24)	10 (24)	9 (24)
Smoking status			
Never/former smoker	56 (71)	29 (69)	27 (73)
Current smoker	1 (1)	1 (2)	0 (0)
BMI (kg/m^2^), mean ± SD	30.3 (2.8)	30.5 (2.9)	30.1 (2.6)
Energy expenditure through physical activity (kcal/day), mean ± SD^a^	515.0 (176.2)	526.8 (204.6)	500.9 (137.1)
Daily dietary consumption, mean ± SD^b^			
Total calories	1729.3 (409.2)	1760.9 (299.0)	1729.3 (409.2)
Fat (% of calories)	31.4 (4.9)	30.3 (5.1)	32.4 (4.4)
Protein (% of calories)	18.8 (3.7)	17.2 (2.7)	20.3 (3.9)
Carbohydrates (% of calories)	50.2 (6.7)	53.5 (6.0)	46.9 (5.6)

^a^Baseline physical activity data available only for 57 participants.

^b^Baseline daily dietary consumption data were available only for 73 participants.

### Effects of interventions on serum adipokine measures

Both interventions resulted in significant weight loss (overall average 3.2 kg [*P* < 0.01]), thus we assessed whether these effects translated into favorable modifications of serum adipokine concentrations. Mean serum adiponectin, leptin and the A/L ratio were examined at baseline, and weeks 34 and 52 for LFD, LCD, and overall (Table 2). There were non-significant decreases in serum adiponectin for both diets, which did not significantly differ by arm. Among those randomized to the LFD, adiponectin decreased 4.1% and 8.5%, comparing weeks 34 and 52 to baseline concentrations (*P* = 0.29 for test of linear trend); while among the LCD group, adiponectin decreased 2.3% and 5.0%, at those time points, respectively (*P* = 0.66 for test of linear trend). The average intervention effects included a 3.3% decrease in adiponectin at week 34 and a 6.8% decrease at week 52; however, the average linear trend in time across diets was not significant for adiponectin (*P* = 0.29). Mean serum leptin concentrations decreased following the LFD and LCD interventions and overall, with no significant differences by intervention arm. Among the LFD group, leptin decreased 30.0% and 23.0% (*P*-values = 0.01 and 0.15, respectively) and among the LCD group, leptin decreased 35.3% (*P* = 0.004) and 19.7% (*P* = 0.28), at weeks 34 and 52, respectively. Overall, the interventions yielded 34.1% and 21.4% lower leptin concentrations at weeks 34 and 52, respectively, than at baseline (*P*-values = 0.0004 and 0.08, respectively). Overall, the change in leptin from baseline to week 52 was not significant, indicating that serum leptin returned to baseline concentrations by the end of the trial. Mean serum A/L ratio increased following the interventions. Among the LFD group, the A/L ratio increased 10.5% and 23.7% (*P* = 0.29 for test of linear trend), and among the LCD group, the A/L ratio increased 19.4% and 44.4% at weeks 34 and 52 (*P* = 0.09 for linear trend). The overall trend in A/L ratio, across groups, was marginally significant (*P =* 0.05) with increases of 16.2% and 35.1% at weeks 34 and 52.

**Table 2:** **Effects of low-fat and low-carbohydrate diets on serum adipokine concentrations, N = 71**

Biomarker	Intervention arm	Baseline	Week 34	Week 52
		Geometric mean (95% CI)	Geometric mean (95% CI)	Geometric mean (95% CI)
	Low-fat	10.41 (8.87–12.22)	9.98 (8.62–11.56)	9.52 (7.83–11.58)
Adiponectin (μg/mL)	Low-carbohydrate	11.03 (9.32–13.05)	10.77 (9.21–12.58)	10.48 (8.50–12.91)
	Overall	10.72 (9.54–12.03)	10.37 (9.31–11.54)	9.99 (8.66–11.52)
Leptin (ng/mL)^a^	Low-fat	28.37 (22.72–35.43)	19.90 (15.73–25.18)	21.85 (16.21–29.45)
	Low-carbohydrate	31.82 (25.34–39.95)	20.60 (15.99–26.54)	25.56 (18.02–36.24)
	Overall	30.05 (25.63–35.23)	19.80 (16.25–24.14)	23.63 (18.78–29.73)
A/L ratio^b^	Low-fat	0.38 (0.29–0.51)	0.42 (0.33–0.54)	0.47 (0.33–0.68)
	Low-carbohydrate	0.36 (0.27–0.49)	0.43 (0.33–0.56)	0.52 (0.35–0.76)
	Overall	0.37 (0.30–0.46)	0.43 (0.35–0.51)	0.50 (0.38–0.64)

NOTE: Mixed models with repeated measurements of natural log transformed serum adipokine concentrations were used.

Overall estimates are averages across the two diets. Estimates for leptin assume a quadratic trend in time. All other estimates assume a linear trend in time. Data presented correspond to geometric means and 95% CIs. No difference in trend by diet was observed for any biomarker.

^a^Significant quadratic trend was observed for serum leptin (*P* = 0.01); no difference observed between baseline and Week 52 serum leptin concentrations (*P* > 0.05).

^b^Average trend in time across diets was borderline significant for serum A/L ratio (*P* = 0.05).

We conducted secondary analyses to determine intervention effects among diet-adherent participants (n = 18; Table 3) and observed slightly different patterns of adipokine change than those observed overall, although these findings were wholly non-significant. While among the LFD group, there appeared to be a decrease in adiponectin following the intervention, among the LCD group adiponectin tended to increase. Conversely, both interventions consistently yielded decreased serum leptin concentrations. The change pattern of the A/L ratio also differed from that observed overall. Similar to the adiponectin observations, among the LFD group, there was a decrease in the A/L ratio, whereas among the LCD group there was an increase.

**Table 3:** **Secondary analysis of the effects of low-fat and low-carbohydrate diets on serum adipokine concentrations among women who were adherent to the dietary component of the intervention, N = 18**

Biomarker	Intervention arm	Baseline	Week 34	Week 52
		Geometric mean (95% CI)	Geometric mean (95% CI)	Geometric mean (95% CI)
	Low-fat	11.93 (8.13–17.50)	10.11 (7.02–14.57)	8.39 (4.85–14.53)
Adiponectin (μg/mL)	Low-carbohydrate	11.06 (7.53–16.25)	11.55 (8.20–16.27)	12.11 (7.69–19.09)
	Overall	11.49 (8.76–15.07)	10.80 (8.41–13.88)	10.08 (7.06–14.40)
Leptin (ng/mL)	Low-fat	28.21 (16.76–47.50)	22.21 (12.80–38.54)	22.97 (7.95–66.37)
	Low-carbohydrate	29.12 (17.24–49.18)	25.34 (14.92–43.04)	20.36 (9.41–44.06)
	Overall	28.66 (19.81–41.48)	24.65 (16.00–37.97)	21.62 (11.23–41.65)
A/L ratio	Low-fat	0.43 (0.23–0.80)	0.42 (0.23–0.76)	0.41 (0.16–1.06)
	Low-carbohydrate	0.38 (0.20–0.71)	0.46 (0.27–0.80)	0.58 (0.27–1.25)
	Overall	0.40 (0.26–0.63)	0.44 (0.29–0.66)	0.49 (0.27–0.90)

NOTE: Mixed models with repeated measurements of natural log transformed serum adipokine concentrations were used. Overall estimates are averages across the two diets. Estimates for leptin assume a quadratic trend in time. All other estimates assume a linear trend in time. Data presented correspond to geometric means and 95% CIs. No difference in trend by diet was observed for any biomarker.

We also examined intervention effects specifically among PA-adherent participants (n = 49; Table 4). We observed no change in serum adiponectin, whereas significant effects on leptin and the A/L ratio were found. Mean leptin concentrations decreased 34.1% (*P* = 0.01) and 27.3% (*P* = 0.09), among the LFD group, and decreased 35.0% and 33.9% (*P*-values = 0.01 and 0.06, respectively) among the LCD group at weeks 34 and 52. Overall, there were 34.1% and 30.7% decreases in leptin at weeks 34 and 52 (*P*-values = 0.002 and 0.01, respectively). Mean A/L ratio increased 13.0% and 35.0% at weeks 34 and 52 among the LFD group (*P* = 0.16 for linear trend), and increased 25.7% and 62.9% at weeks 34 and 52, among the LCD group (*P* = 0.05 for linear trend). Overall, there were 18.4% and 44.7% increases in the A/L ratio at weeks 34 and 52 (*P* = 0.02 for linear trend).

**Table 4:** **Secondary analysis of the effects of low-fat and low-carbohydrate diets on serum adipokine concentrations among women who were adherent to the physical activity component of the intervention, N = 49**

Biomarker	Intervention arm	Baseline	Week 34	Week 52
		Geometric mean (95% CI)	Geometric mean (95% CI)	Geometric mean (95% CI)
	Low-fat	9.72 (8.11–11.65)	9.55 (8.11–11.24)	9.36 (7.61–11.52)
Adiponectin (μg/mL)	Low-carbohydrate	10.86 (8.90–13.24)	10.66 (8.91–12.76)	10.44 (8.31–13.13)
	Overall	10.27 (8.98–11.75)	10.09 (8.94–11.39)	9.89 (8.47–11.54)
Leptin (ng/mL)^a^	Low-fat	25.93 (20.38–32.98)	17.07 (13.54–21.52)	18.85 (14.13–25.16)
	Low-carbohydrate	31.56 (24.61–40.47)	20.52 (15.80–26.64)	20.87 (14.47–30.10)
	Overall	28.61 (24.06–34.01)	18.85 (15.30–15.30)	19.83 (15.71–25.04)
A/L ratio^b^	Low-fat	0.40 (0.30–0.54)	0.46 (0.36–0.59)	0.54 (0.38–0.77)
	Low-carbohydrate	0.35 (0.26–0.49)	0.44 (0.34–0.58)	0.57 (0.38–0.85)
	Overall	0.38 (0.30–0.47)	0.45 (0.37–0.54)	0.55 (0.42–0.72)

NOTE: Mixed models with repeated measurements of natural log transformed serum adipokine concentrations were used. Overall estimates are averages across the two diets. Estimates for leptin assume a quadratic trend in time. All other estimates assume a linear trend in time. Data presented correspond to geometric means and 95% CIs. No difference in trend by diet was observed for any biomarker.

^a^Borderline significant quadratic trend was observed for serum leptin (*P* = 0.05); no difference observed between baseline and Week 52 serum leptin concentrations (*P* > 0.05).

^b^Average trend in time across diets was significant (*P* = 0.02). Significant difference observed between baseline and Week 52 for the A/L ratio for low-carbohydrate diet (*P* = 0.05).

## Discussion

Currently, overweight and obese premenopausal women, who have an increased likelihood of sustaining higher body weight after menopause, lack effective, practical methods for reducing their breast cancer risk. Attractive strategies for risk reduction may include lifestyle modifications. In the present study, we hypothesized that LFD and LCD dietary patterns could favorably modify serum adipokine concentrations (*i.e.*, increase adiponectin, decrease leptin, and increase the A/L ratio) through weight reduction, and therefore, promote the prevention of obesity-related breast cancer among premenopausal women. Our findings demonstrated that, overall the LFD and LCD interventions yielded significant reductions in serum leptin, while the modifications of adiponectin and the A/L ratio were statistically non-significant. Notably, among women who were adherent to the LCD intervention, adipokine profiles improved in the directions that are hypothesized to be protective.

Several dietary trials have evaluated the effects of LFD and/or LCD interventions on adipokine concentrations (de Luis et al. 2007; Bluher et al. 2012; Befort et al. 2012; Scott et al. 2013; Giannopoulou et al. 2005; Friedenreich et al. 2011; Sacks et al. 2009; Davis et al. 2009; Foster et al. 2003; Samaha et al. 2003; Brehm et al. 2003; Yancy et al. 2004; Shai et al. 2008; Bradley et al. 2009; Reed et al. 2010; Ata et al. 2010; Ong et al. 2009; Harvie et al. 2011), but few have focused on premenopausal women (Reed et al. 2010; Ata et al. 2010; Ong et al. 2009; Harvie et al. 2011). In our study, overweight and obese premenopausal women were randomized to a LFD or a LCD plus PA for a period of 52 weeks. While adherence to these interventions was fairly low, there tended to be larger decreases in serum leptin among diet-adherent participants. One study (Reed et al. 2010) examining the effects of a 4-month calorie-restricted diet of similar composition to the LFD examined herein, combined with aerobic exercise among premenopausal women, demonstrated no significant change in circulating adiponectin and a 52% decrease in leptin among normal weight to obese premenopausal women. They concluded that the substantial reduction in leptin was exercise-induced (Reed et al. 2010) and due to significant fat mass reductions over the course of their intervention trial. Our findings support this hypothesis given that we too observed significant reductions in leptin (approximately 30%), particularly among PA-adherent participants. However, the smaller reduction in leptin observed in our study could be attributable to the lower intensity of the walking-based PA to which women were prescribed, translating into smaller reductions in fat mass (which we did not examine). Another study (Ata et al. 2010), examining the effects of a 10-week LCD (with the same macronutrient composition as the LCD studied herein) plus a walking intervention among overweight and obese premenopausal women, demonstrated 10% higher adiponectin and 6% lower leptin post-intervention. A recent study (Harvie et al. 2011) examined the effects of a 6-month trial of intermittent and continuous calorie-restricted diets without exercise among premenopausal women and found that these diets yielded significant reduced leptin (approximately 40%) and increased the A/L ratio (approximately 20%). Another study (Ong et al. 2009) examined the effects of a 1-month calorie-restricted intervention (62% carbohydrates, 26% protein, and 12% fat), without exercise, on adipokine concentrations and adipokine expression profiles among overweight and obese premenopausal women. They found that post-intervention adiponectin and leptin concentrations were 3% and 60% lower, respectively (Ong et al. 2009). Interestingly, this study (Ong et al. 2009) also identified several genes involved in metabolic pathways (*e.g.*, glycolysis and lipid synthesis) with altered gene expression in breast and adipose tissues post-intervention. These findings (Ong et al. 2009), as well as findings from our previous work (Llanos et al. 2012), highlight the importance of understanding adipokine concentrations within local breast tissues and their role in breast cancer risk and progression.

While the precise mechanism(s) involved in the potentially protective effects of caloric-restriction are unclear, several studies have concluded that dietary interventions of this type are effective for weight loss, particularly when they are combined with exercise (Ata et al. 2010; Befort et al. 2012; Reed et al. 2010; Scott et al. 2013), and therefore, would be effective for obesity-related cancer prevention (Harvie et al. 2005; Kawai et al. 2010; Trentham-Dietz et al. 2000), as well as for improving breast cancer outcomes (Scott et al. 2013; Befort et al. 2012). Our findings of favorable modification of adipokine profiles, particularly leptin, among women who were adherent to the intervention, support this hypothesis. Previous studies tend to show a positive association between circulating leptin and breast cancer (Minatoya et al. 2013; Liu et al. 2013; Ye et al. 2014; Tian et al. 2007; Tworoger et al. 2007; Mantzoros et al. 2004; Miyoshi et al. 2003), although the data for premenopausal women have varied. Additional studies have provided evidence to support a role for an inverse association between the A/L ratio and breast cancer risk and prognosis (Chen et al. 2006; Goodwin et al. 2012), suggesting the balance of adiponectin and leptin, rather than either biomarker alone, may also be clinically significant. Further study to help explain the underlying mechanisms that may be responsible for the beneficial adipokine effects of calorie-restricted interventions are necessary.

Notably, we observed stronger percent change in leptin, compared to those in adiponectin and the A/L ratio, following the intervention period. Similarly, findings from other dietary intervention trials have suggested that calorie restricted diets, particularly the LCD variety, in combination with exercise, produce rapid weight loss (Ata et al. 2010; Befort et al. 2012; Reed et al. 2010; Scott et al. 2013), which results in larger reductions in circulating leptin concentrations. This is suggestive that the effects of the macronutrient composition may be dependent on the extent of weight loss induced by the dietary pattern. Consequently, reductions in circulating leptin, with or without maintained weight loss, could elicit considerable breast cancer preventive effects. Leptin promotes cellular growth and proliferation, induces signaling pathways involved in survival of breast epithelial cells (Laud et al. 2002; Dieudonne et al. 2002), and promotes angiogenesis (Cao et al. 2001). Furthermore, assuming the reported associations between circulating leptin concentrations and breast cancer risk and adverse events in breast cancer survivors (Goodwin et al. 2012) are accurate, interventions targeting leptin may be promising for breast cancer prevention and control efforts in the future. While our findings support the hypothesis that caloric-restriction may be an attractive method for obesity-related cancer prevention through altering the leptin signaling pathway (and possibly through mechanisms not studied here), the clinical significance of the favorable changes observed herein is unclear. Future studies involving larger samples are required to confirm the effects in the general population.

There were various strengths of this study. First, the randomized nature of the dietary intervention trial and the addition of a PA component strengthened the study. In addition, most studies have examined the effects of PA and LFD and/or LCD interventions for a period of six months or shorter (de Luis et al. 2007; Befort et al. 2012; Scott et al. 2013; Giannopoulou et al. 2005; Yancy et al. 2004; Reed et al. 2010; Ata et al. 2010; Ong et al. 2009), whereas our study period was one year. Our examination of the intervention effects on weight loss as well as biomarkers previously shown to be associated with obesity and breast cancer risk was also a strength. Relatedly, our use of highly reproducible immunoassays to measure serum adipokine concentrations as well as their measurement at several time points over the course of the study was an additional strength. Furthermore, our use of mixed models for the analysis of treatment and time effects (under the assumption that missing data were random) allowed us to use all of the available data, further strengthening our findings.

This study also suffered from certain limitations that should be considered. The small sample size (41 and 38 women randomized to LFD and LCD, respectively), which included only overweight and obese premenopausal women, limited our power to observe differences in biomarker concentrations between diets as well as limited the generalizability of our findings. Secondly, the relatively high losses to follow-up and low rates of adherence to the LFD and LCD diets may have contributed to our finding small differences in adipokine concentrations between the interventions. While rates of adherence to the PA component of the intervention were higher, likely as a result of participants wearing pedometers, this too could have tempered our findings given that PA was self-monitored. Similarly, the use of self-reported dietary consumption through 7-day diet recalls may be have been a limitation given that complete data were often not collected. Additionally, it is likely that participants’ dietary patterns may have been altered and/or reported inaccurately due to the burden of recalling their consumption and possibly due to social influence. Finally, measurement of only total adiponectin, as opposed to other isoforms, which could have been modified by the interventions, may have affected our findings.

In summary, findings from this small randomized trial support LCD and LFD dietary interventions as potentially attractive methods for obesity-related breast cancer prevention, particularly through favorable modification of leptin among premenopausal women. Development of effective and practical interventions involving sustainable dietary and PA changes that have significant, favorable effects on adiponectin, leptin and the A/L ratio (and other related biomarkers) would be important next steps in this line of research.

## Abbreviations

A/L: Adiponectin-to-leptin ratio
BMI: Body mass index
LCD: Low-carbohydrate diet
LFD: Low-fat diet
PA: Physical activity
WHR: Waist-to-hip ratio.
